# The Influence of Gel Preparation and Thermal Treatment on the Optical Properties of SiO_2_-ZnO Powders Obtained by Sol–Gel Method

**DOI:** 10.3390/gels8080498

**Published:** 2022-08-11

**Authors:** Oana-Cătălina Mocioiu, Cristina Maria Vlăduț, Irina Atkinson, Veronica Brătan, Ana-Maria Mocioiu

**Affiliations:** 1 Institute of Physical Chemistry Ilie Murgulescu of the Romanian Academy, 202 Splaiul Independenţei, 060021 Bucharest, Romania; 2 National R&D Institute for Non-Ferrous and Rare Metals, 102 Biruinţei Blvd, 077145 Pantelimon, Romania

**Keywords:** SiO_2_-ZnO, sol–gel method, FTIR, thermal treatment, UV-VIS

## Abstract

The effect of gel preparation and heat treatment on the structural and optical properties of SiO_2_-ZnO materials prepared by the sol–gel method was investigated. Zinc acetate dehydrate, TEOS (tetraethylortosilicate), ethanol, distillated water and HCl were used as a starting material, solvent and catalyst, respectively. Four powders (G1–G4) were prepared in different ways from the starting materials mentioned above. The method of adding Zn precursors during the synthesis differed from one another. For the G1 synthesis, only Zn acetate powder was employed; for the G2 synthesis, Zn acetate was dissolved in distilled water; and for the G3 synthesis, Zn acetate was dissolved in ethanol. When synthesizing G4, TEOS was added last, after Zn acetate had been combined with water and ethanol. The SiO_2_-ZnO materials were dried at 200 °C and then heat-treated at 700 °C and 900 °C. All samples were investigated by X-ray diffraction and infrared spectroscopy in order to investigate their structure. SEM measurements were performed to investigate the morphology of materials. Optical properties were influenced by gel preparation and heat treatments. A reflectance of over 60% was obtained for G3 and G4 powders, while for G1 and G2, the reflectance was below 30%. In conclusion, synthesis steps and heat treatment can control the structure and properties of the powders.

## 1. Introduction

Materials in the system SiO_2_-ZnO have been attracting considerable attention due to their optical properties such as green-yellow band emissions and photoluminescence [1,2,3,4], as well as the applicability in the sensor field [5,6], their antibacterial activity [7,8] and anticorrosive resistance [9]. At ambient temperature, pure ZnO is a semiconductor with a large direct band gap of roughly 3.3 eV. ZnO films are transparent across the visible spectrum [10].

There are several studies on SiO_2_-ZnO materials reported in the literature, some of which are presented below. Our target was to obtain sol–gel SiO_2_-ZnO powders and to investigate the effect of gel synthesis and heat treatment on their structural and optical properties. Sol–gel was used to create ZnO nanocrystallites that were contained in mesoporous silica with a ZnO:SiO_2_ ratio of 20:80 [4]. It was found that the SiO_2_ matrix was a very successful coating medium that only produced an amount of ZnO nanoparticles with quite small particle sizes (1.42–1.47 nm). Due to the efficient integration of the nanoparticles in the SiO_2_ matrix, the growth in granule size in the ZnO-SiO_2_ composite films stopped at 773 K. The optical properties of nanoparticles are extremely limited indicating high stability after annealing treatments at 773 K post-deposition. XRD indicated the wurtzite structure on predominant axe (002). A variation in the range 4.23–4.29 eV occurred with the variation in the particle 1.42–1.47 nm. When one of the films was annealed at 1073 K for 7 h, the prohibited band abruptly decreased from 4.3 eV to 3.33 eV (bulk value) as a result of the quick grain development. This resulted in high UV luminescence for the ZnO nanoparticles in the silica matrix after annealing at 773 K for 30 min in an oxygen-rich environment [4].

Based on a ZnO-SiO_2_ composite film obtained using the sol–gel method, Wang [5] created an NH_3_ gas sensor with a surface acoustic wave. The sensor with the highest detection capabilities has a maximum frequency shift of 1.132 kHz below 10 ppm ammonium gas and a ratio of ZnO:SiO_2_ = 1:2. By altering the molar ratio of ZnO and SiO_2_, the sensor’s sensitivity can be raised. A significant response can also be influenced by the presence of water on the surface and the creation of Si-O-H bonds since water can absorb more ammonia molecules. The less crystallized film was the best.

Ali [6] looked at how heat treatment affected the structural and optical characteristics of ZnO:SiO_2_ nanocomposite films. The sol–gel technique was used to create thin films on Si substrates. Tetraethoxysilane, water, C_2_H_5_OH, and HCl had an equimolar ratio of 1:4:5:0.25. Zinc acetate Zn(CH_3_COO)_2_ was added to silica sol while being intensively stirred for three hours in order to produce composites of 5 wt percent ZnO:SiO_2_. Treatment at high temperatures results in an increase in the c-axis orientation of ZnO. There were two optical band gaps found, at about 3.0 eV and 4.2 eV. The first peak’s origin may be associated with ZnO, whereas the second peak’s origin may be associated with SiO_2_. ZnO:SiO_2_ nanoparticles grow in size as the temperature rises. A lower detection limit and good sensitivity were displayed by the ZnO:SiO_2_ film. The cyclic test demonstrated that the sensor for liquid–solid chemical detection was stable throughout time [6].

Antireflective coatings often consist of single- or multi-layer structures. High reflection causes light loss, which lowers the efficiency of optoelectronics including photodetectors, solar cells, and light-emitting devices [7,8,9,10,11]. To reduce reflection and enhance performance in optical and optoelectronic applications, antireflection films are necessary. A number of methods, including photolithography [8], imprinting [7], etc., can be used to create thin films. These technologies make use of previously created high-purity oxides. Excellent production rates, low energy costs, and high material purity are all benefits of the sol–gel process.

For the deposition of meso- and microporous silica-based films, the sol–gel technique provides a quick and inexpensive procedure [12]. ZnO nanoparticles having a hexagonal shape and a desired orientation (100) are very effectively incorporated into the silica matrix in ZnO:SiO_2_ films with crack-free mesoporous morphology. SiO_2_:ZnO had an equivalent molar ratio of 76:24, and TEOS, C_2_H_5_OH, H_2_O, HCl, and CTAB had molar ratios of 1:25:7:0.14:0.05. Using the dip-coating process, the films were applied to a Corning 1737 glass substrate at withdrawal speeds of 10, 20, and 30 cm/min. With optical transmission ranging from 75% to 95% in the visible and infrared, the films are extremely transparent. The computed Eg values are much higher than those of the typical ZnO bulk material (3.3 eV) and the band gap of ZnO:SiO_2_ composite decreases with increase in thickness and deposition speed (from 5.26 eV to 4.18 eV) [12]. The potential for porous ZnO:SiO_2_ films as catalysts and gas-sensing materials is tremendous [12].

He et al. looked into the photoluminescence behavior of ZnO particles embedded in sol–gel silica at ambient temperature. Approximately, 3.25 eV (ultraviolet (UV)), 2.8 eV (blue), and 2.45 eV (green) ZnO emissions were found. Solutions of ethanol, zinc acetate (ZA), HNO_3_, and tetraethoxysilane (TEOS) were utilized as precursors. According to the XRD results, crystalline ZnO was produced at temperatures above 800 °C, and several peaks associated with Zn_2_SiO_4_ were seen [13].

Mohamed et al. prepared ZnO-SiO_2_ mixed oxides via a sol–gel approach using TEOS and Zn (NO_3_)_2_·6H_2_O. They exhibit several crystalline peaks mainly composed of the ZnO crystallites’ phase. The obtained xerogel material was used for photocatalytic degradation of methylene blue dye. The optimal condition was to maintain a 30:70 molar ratio for the ZnO:SiO_2_ at room temperature for 60 min [14].

By developing a gel-drying technique and simultaneously controlling ZnO grain formation by optimizing the molar ratio of ethylene glycol (EG)/nitrates in its precursors, Chao Yang presented a simple sol–gel strategy for the manufacture of ZnO/SiO_2_ adsorbents for effective H_2_S removal. The created adsorbent has a high H_2_S removal capacity of 108.9 mg S/g sorbent due to the well-dispersed ZnO nanograins (57 wt percent ZnO loading) that were incorporated into a SiO_2_ matrix [15].

Due to the lack of information on the amorphous SiO_2_-ZnO powders produced by the sol–gel method and the fact that a material’s properties are strongly influenced by its microstructure, it is necessary to investigate how the thermal treatment and the order in which the precursors are added during the synthesis influences the structure and morphology of the final product.

In this study, we looked at how heat treatment and gel preparation affected the structural and optical characteristics of sol–gel-prepared SiO_2_-ZnO materials. The produced powders can be utilized in additive manufacturing to create dense or porous films for use in optoelectronics and catalysis. Based on the literature presented above, the novelty of the paper consists of obtaining different optical properties for non-crystalline solids as 90%SiO_2_-10%ZnO by modifying the synthesis steps. 

## 2. Materials and Methods

### 2.1. Synthesis 

In this article, we looked at how the different reaction steps affected the characteristics of the gels that were produced. With TEOS (tetraethyl ortosilicate) [Si(OC_2_H_5_)_4_], zinc acetate dehydrate, ethanol, distillated water, and HCl as a starting point, the solutions are made using the sol–gel method. All solutions had the following molar ratio: C_2_H_5_OH:TEOS:H_2_O:HCl = 10:1:3:0.03. To obtain non-crystalline (vitreous) materials, high dilution was employed.

Synthesis 1: Hydrochloric-acid-acidified water was added after mixing TEOS and ethanol for 30 min. The zinc acetate dehydrate powder was dissolved in the solution and agitated for an additional 30 min.

Synthesis 2: Hydrochloric-acid-acidified water was added after mixing TEOS and ethanol for 30 min. After that, it was filled with a water-dissolved zinc acetate dehydrate solution, which was agitated for 30 min.

Synthesis 3: Involved mixing TEOS and ethanol for 30 min before adding and stirring hydrochloric-acid-acidified water. After 30 min of stirring, a solution of ethanol and zinc acetate dehydrate was poured on top.

Synthesis 4: TEOS was added to a solution of zinc acetate dehydrate, water, HCl, and ethanol after it had been agitated for 30 min.

The ageing time of the solutions was 18 h and they were dried at 200 °C in order to obtain the gels noted G1, G2, G3 and G4. A part of each solution was kept at room temperature to determine the gelling time. Solution 1 gelled in two weeks and became solid after one month. Solution 2 and 3 remained liquid and transparent after three months and became solids in one year. Solution 4 became a transparent gel after 1 month and solidified after three months. The final concentration for all syntheses is 90 mol% SiO_2_ and 10 mol% ZnO. The bulk gels were dried in an oven, grounded and heat-treated at 700 °C and 900 °C for 2 h. 

**Figure 1:**
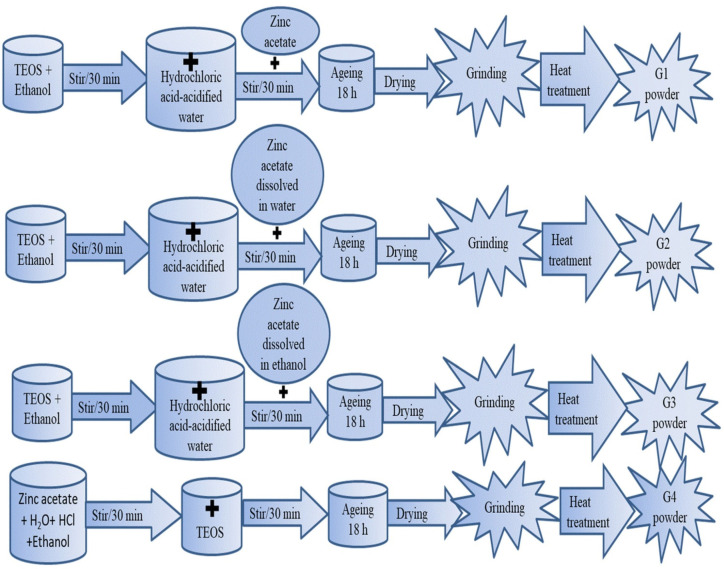
The synthesis schemes of G1–G4 powders.

In Figure 1, the synthesis schemes of G1–G4 powders are presented.

### 2.2. Characterization

Gels and treated powders were characterized by Fourier-transform infrared spectroscopy (FTIR); X-ray diffraction (XRD); scanning electron microscopy (SEM) and diffuse reflectance UV-VIS.

Using a Rigaku Ultima IV diffractometer (Rigaku Corporation, Tokyo, Japan) in parallel beam geometry with CuK radiation (wavelength 1.5406 Å) in the 2θ range between 10 and 70 with a speed of 2°/min and a step size of 0.02°, the gels and treated powders were subjected to X-ray diffraction (XRD). Thermo Fisher Scientific Inc., Waltham, MA, USA, Nicolet 6700 instrument was used to record the Fourier-transform infrared (FTIR) spectra of the gels and treated powders in the 400–4000 cm^−1^ domain with a sensibility of 4 cm^−1^. One milligram of the finely powdered sample was combined with 200 milligrams of KBr and pressed into a clear pellet. The morphology of the gels was studied using scanning electron microscopy (SEM) using a Quanta 250 (fitted with EDAX detector, FEI Company, Eindhoven, The Netherlands) model of microscope. In order to prepare, the sample was immobilized using double-sided carbon tape with no coating. In order to study optical properties, UV-VIS spectroscopy was used. With the aid of an integrating sphere-equipped spectrophotometer, a Perkin Elmer Lambda 35, diffuse reflectance UV-VIS spectra were acquired. With spectralon as a reference, measurements were made on powders in the wavelength range of 1100–200 nm. The Kubelka–Munk function, F(R), was used to translate the reflectance values into absorption spectra.

## 3. Results and Discussion

**Figure 2:**
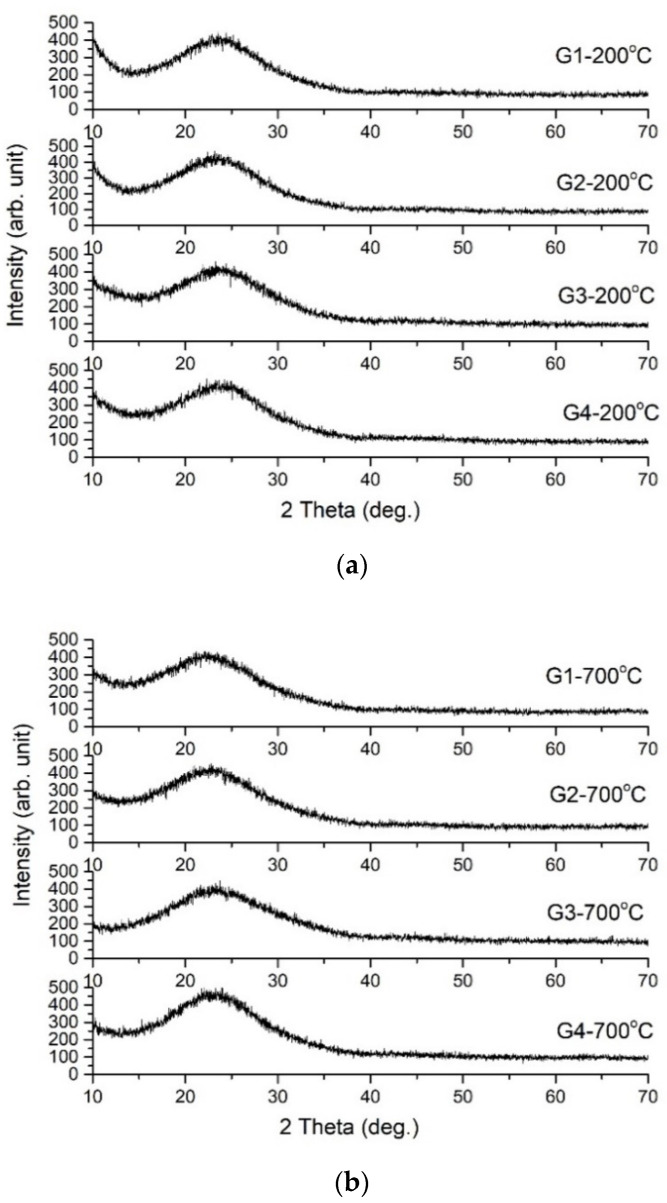

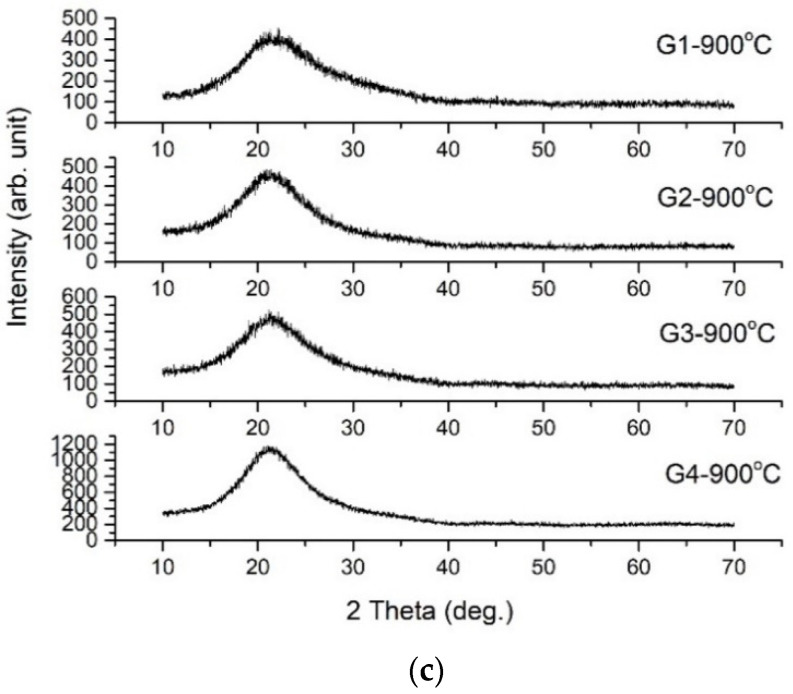
XRD patterns of powders G1–G4 treated at (**a**) 200 °C, (**b**) 700 °C and (**c**) 900 °C.

In Figure 2a–c, the X-ray diffraction patterns for powders thermally treated at 200 °C, 700 °C and 900 °C, are presented. All powders have non-crystalline structures as can be seen in the figures. Even after heat treatment at 900 °C, no crystallization of any compound is observed.

**Figure 3:**
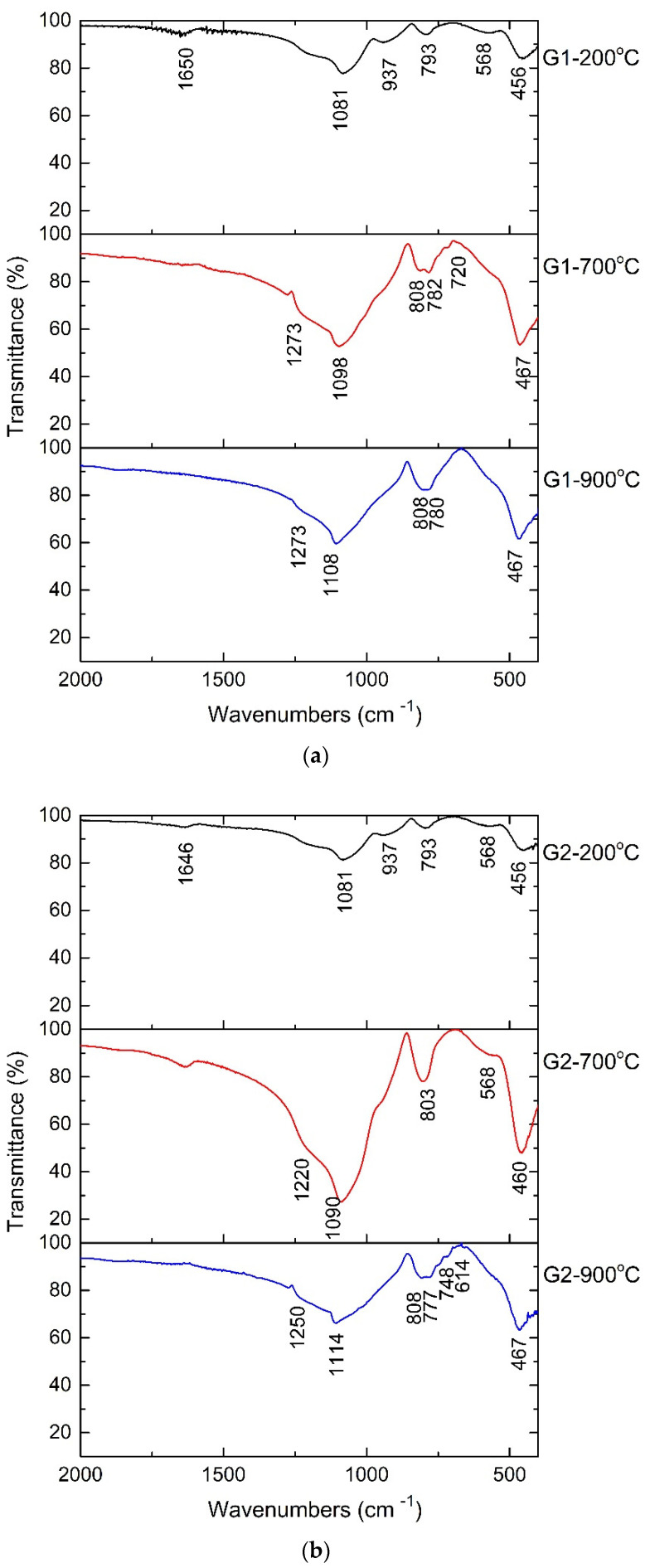

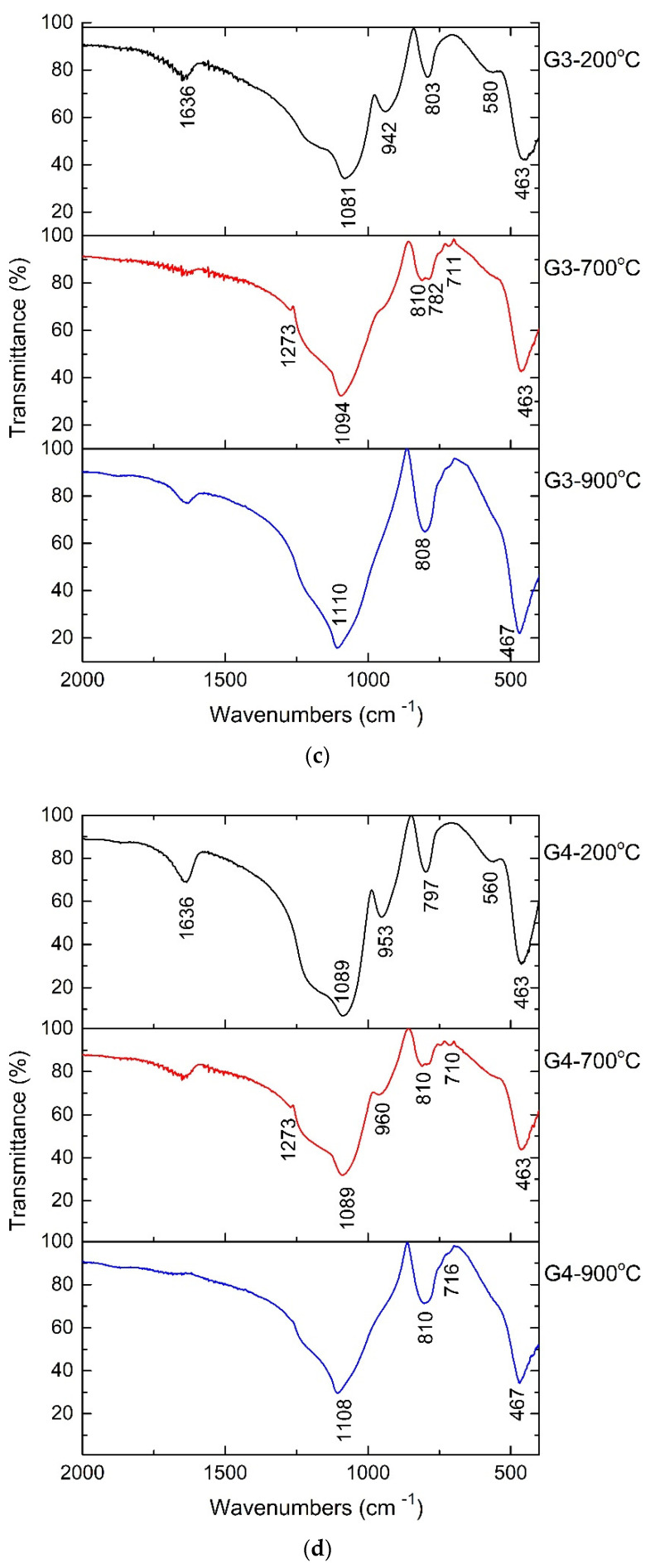
FTIR spectra of powders heat treated at 200 °C, 700 °C and 900 °C: (**a**) G1; (**b**) G2; (**c**) G3; (**d**) G4.

The FTIR spectra for treated powders are shown in Figure 3. The bands that are typical of Si-O vibrations are visible in the recorded spectra and are identical to those that have been described in the literature [16,17,18,19,20,21]. Small bands at 560 cm^−1^ or 568 cm^−1^ may be attributed to the vibration of the Si-O-Zn bonds, whereas the main bands at 1273 cm^−1^, 1080 cm^−1^, 1114 cm^−1^, 793 cm^−1^, and 467 cm^−1^ are assigned to Si-O-Si bonds in networks and chains. Band at 937 cm^−1^ is characteristic of isolated [SiO_4_] tetrahedra. Due to its wide bands, the FTIR spectrum reveals the formation of a vitreous material. In the non-crystalline solids, the structural units are ordered at small distances such as isolated SiO_4_^4−^ tetrahedra (structural unit); pairs of tetrahedra, chains of tetrahedra, double chains (rings) of tetrahedra and network (3D). The bands between 800 and 900 cm^−1^ are characteristic to stretching motions of Si-O in isolated tetrahedra with 4 non-bridging oxygen/tetrahedra. Bands at 943 and 1094 cm^−1^ are characteristic of Si-O-Si vibrations in chains and sheet (ring) of tetrahedra with 1 or 2 non-bridging oxygen. The framework vibrations were observed in 1108–1273 cm^−1^ domain. Si-O and Zn-O bond’s band position of vibration can be seen in the spectrum, but they cannot be attributed to crystalline forms. As a result, the powders also have a vitreous structure. The O-H vibration of the surface-absorbent water is attributed to the bands at 1650 cm^−1^ and 1636 cm^−1^. In Table 1 are presented all the bands for initial and treated powders and their assignations.

The amorphous powders show good transmittance. G1 powder shows the best transmittance value, while the structure of G4 powder is the most ordered of all the studied samples.

**Figure 4:**
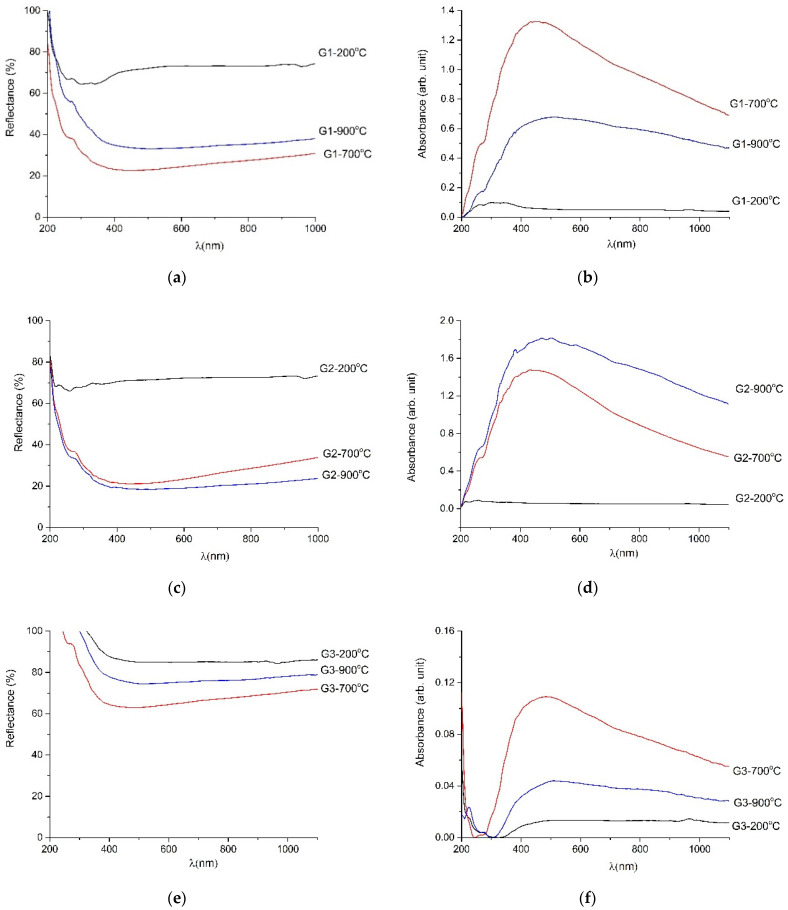

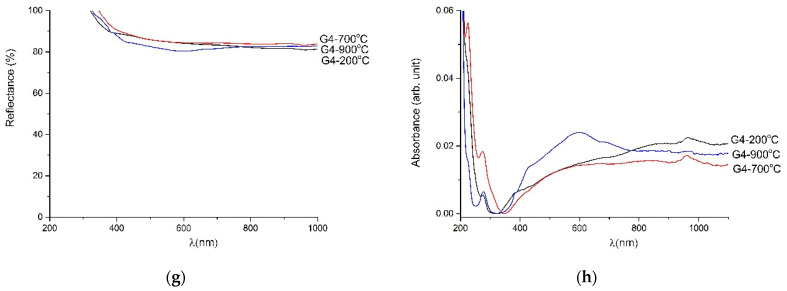
UV_VIS spectra listed as: (**a**) reflectance spectra of heat-treated G1 powders; (**b**) absorbance spectra of heat-treated G1 powders; (**c**) reflectance spectra of heat-treated G2 powders; (**d**) absorbance spectra of heat-treated G2 powders; (**e**) reflectance spectra of heat-treated G3 powders; (**f**) absorbance spectra of heat-treated G3 powders; (**g**) reflectance spectra of heat-treated G4 powders; (**h**) absorbance spectra of heat-treated G4 powders.

Figure 4 shows the reflectance spectra of powders produced in different synthesis modes (1–4). For G1–G4 at 200 °C all reflectance values were above 6o% from 200 to 1100 nm wavelength (λ). Figure 4 a,c show the decrease in reflectance below 30% for the G1 and G2 heat-treated powders at 700 °C and 900 °C. The reflectance below 390 nm wavelength was decreased due to the intrinsic absorption of ZnO as Chang showed in reference [7]. So, the synthesis for G1 and G2 powders is suitable for materials used for printing antireflective coatings. The reflectance was above 60% for heat-treated G3 and G4 powders. They are highly reflective materials and can be ideal for UV and lighting systems used in water disinfection. On the other part, the absorbance spectra of powders present one absorption band in visible domain: at 450 nm (violet region) for G1, G2 and G3 treated at 700 °C and 900 °C; and at 600 nm (orange region) for G4-900 °C. In the case of powders obtained by synthesis 1, synthesis 2 and synthesis 3 absorption increases after heat treatment at 700 °C and 900 °C, which means that the transmittance decreases. For non-crystalline materials, the high transmittance is desired. In conclusion, powders such as G1-900 °C, G2-700 °C, G3-900 °C and G4-700 °C with lower absorbance and higher transmittance are desirable for solar cell coatings. 

Table 2 gives the band gap values estimated from Tauc plot and the Klubelka–Munk relation. The values vary between 1.2 eV for G1 heat-treated at 900 °C and 5.9 eV for G4 heat-treated at 900 °C.

**Figure 5:**
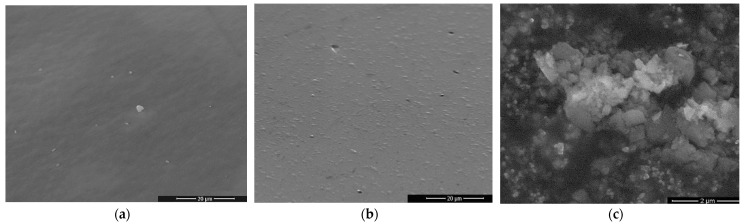
SEM images of heat-treated powders: (**a**) G1-900 °C (×4000), (**b**) G2-900 °C (×4000) and (**c**) G4-900 °C (×20,000).

Figure 5 shows the morphology of gels G1, G2 and G4 after heat treatment at 900 °C. Powders G1 and G2 have a matrix morphology specific to vitreous materials. In Figure 5c, an agglomeration on nanostructures can be seen indicating the beginning of nucleation of G4-900 °C powder. The observation on the morphology of the powders is in agreement with their structure determined by XRD and FTIR spectroscopy.

## 4. Conclusions

In conclusion, the structure and properties of the powders can be controlled by synthesis steps and heat treatment. XRD, FTIR and SEM measurements identified the non-crystalline structure and appropriate morphology for all samples. In the powders’ structure, the structural units SiO_4_^4−^ are present as isolated tetrahedra, chains of tetrahedra or SiO_2_ network but their order is at short distances. All the powders are non-crystalline. The samples with chains and network in the structure present higher band gap values and better reflectance in UV-VIS. Due to a reflectance below 30%, heat-treated G1 and G2 powders are suitable materials for printing of antireflective coatings. The over 60% reflectance of heat-treated G3 and G4 powders may be good for UV and lighting systems used in water disinfection.

## Figures and Tables

**Table 1:** Main bands in FTIR spectra of all powders.

Powder	Bands’ Positions (cm^−1^)								
G1 200	456	568		793	937	1081			1650
G1 700	467		720	782 806		1098		1273	
G1 900	467			780 808		1108		1273	
G2 200	456	568		793	937	1081			1646
G2 700	460	568		803		1090		1220	
G2 900	467	614	748	776 808			1114	1250	
G3 200	463	580		803	942	1081			1636
G3 700	463		711	782 810		1094		1273	
G3 900	467			808			1110		
G4 200	463	560		797	953	1089			1636
G4 700	463		710	810	960	1089		1273	
G4 900	467		716	810			1108		
Assignations									
	Si-O bond in isolated SiO_4_^4−^	Si-O-Zn bonds	Si-O-Si bonds	Isolated SiO_4_^4−^	Chains of units	Chains of units	SiO_2_ network	SiO_2_ network	Si-O-H

**Table 2:** Band gap values.

Treatment Temperature	Band Gap Values (eV)			
	G1	G2	G3	G4
200 °C	2.70	3.02	5.80	5.85
700 °C	1.30	1.46	5.80	5.82
900 °C	1.20	1.50	5.15	5.90
